# Differential Downregulation of E-Cadherin and Desmoglein by Epidermal Growth Factor

**DOI:** 10.1155/2012/309587

**Published:** 2012-01-16

**Authors:** Miquella G. Chavez, Christian A. Buhr, Whitney K. Petrie, Angela Wandinger-Ness, Donna F. Kusewitt, Laurie G. Hudson

**Affiliations:** ^1^Division of Bioengineering, Department of Physiology, University of California San Francisco, San Francisco, CA 94158, USA; ^2^College of Pharmacy, University of New Mexico, MSC 09 5360, Albuquerque, NM 87131, USA; ^3^Department of Animal Science, University of California, Davis, CA 95616, USA; ^4^Department of Pathology, School of Medicine, University of New Mexico, MSC 08 4640, Albuquerque, NM 87131, USA; ^5^Science Park Research Division, Department of Carcinogenesis, University of Texas, M.D. Anderson Cancer Center, Smithville, TX 78957, USA

## Abstract

Modulation of cell : cell junctions is a key event in cutaneous wound repair. In this study we report that activation of the epidermal growth factor (EGF) receptor disrupts cell : cell adhesion, but with different kinetics and fates for the desmosomal cadherin desmoglein and for E-cadherin. Downregulation of desmoglein preceded that of E-cadherin *in vivo* and in an EGF-stimulated *in vitro* wound reepithelialization model. Dual immunofluorescence staining revealed that neither E-cadherin nor desmoglein-2 internalized with the EGF receptor, or with one another. In response to EGF, desmoglein-2 entered a recycling compartment based on predominant colocalization with the recycling marker Rab11. In contrast, E-cadherin downregulation was accompanied by cleavage of the extracellular domain. A broad-spectrum matrix metalloproteinase inhibitor protected E-cadherin but not the desmosomal cadherin, desmoglein-2, from EGF-stimulated disruption. These findings demonstrate that although activation of the EGF receptor regulates adherens junction and desmosomal components, this stimulus downregulates associated cadherins through different mechanisms.

## 1. Introduction

During cutaneous wound repair, epidermal cells at the wound margin undergo phenotypic and functional changes including increased proliferation, migration, and a partial epithelial-to-mesenchymal transition (EMT) characterized by disruption of cell : cell junctions, changes in cell : substrate adhesion, and retraction and reorganization of the cytoskeleton [1–3]. Kalluri and Weinberg proposed three sub-classifications of EMT with type 2 EMT associated with wound healing, tissue regeneration, and organ fibrosis [4]. An essential aspect of type 2 EMT is that once repair is achieved epithelial phenotype and tissue integrity is restored. A hallmark of successful wound repair is the reestablishment of cell : cell junctions and barrier function, thereby illustrating dynamic modulation of adherens junctions and desmosomes during wound reepithelialization. In mouse epidermis, a decrease in E-cadherin is evident 3 days after wounding in full-thickness incisional or excisional wound models with subsequent protein restoration upon wound closure [5]. Blocking E-cadherin function with antibody caused uneven wound margins and disruption of the reorganizing actin cytoskeleton in mouse epidermis [6], demonstrating the importance of E-cadherin in wound repair. Similarly, the dissolution of hemidesmosomes, which connect the cell to the extracellular matrix, and desmosomes responsible for cell : cell contacts occurs during wound healing [7, 8] but little is known about modulation of desmosomal cadherins during wound repair.

A number of mechanisms have been reported to regulate the assembly and disassembly of adherens junctions and desmosomes in cells including phosphorylation of junctional components [9–12], cadherin cleavage [13–18], and endocytic mechanisms [19–26]. Certain signaling molecules such as PKC-*α* are expressed and activated at the wound margin and contribute to the loss of desmosomal adhesion [27]. The epidermal growth factor (EGF) receptor is another important regulator of wound repair. EGF receptor expression is elevated at the leading edge of healing wounds in mice [28, 29] and humans [29], and EGF stimulates wound repair *in vivo* [30–32]. Furthermore, keratinocyte migration *in vitro* and epithelial outgrowth *in vivo* are decreased in EGF receptor null skin [30] indicating that EGF receptor activity is important for optimal wound repair. Activation of the EGF receptor also induces expression of transcriptional regulators of EMT such as Snai2 and regulates junctional assembly and disassembly in keratinocytes [12, 33–38]. It remains unclear how activation of these signaling pathways affect the junctional stability of both desmosomes and adherens junctions to facilitate reepithelialization.

In this study, we investigated the effects of EGF receptor activation on the fate of E-cadherin and desmoglein-2, a desmosomal cadherin that is elevated in healing wounds [39]. We find that downregulation of desmoglein precedes that of E-cadherin *in vivo* and in EGF-stimulated *in vitro* wound reepithelialization models. Importantly, we find evidence for differential regulation of adherens junctions and desmosomal complex proteins as a consequence of EGF receptor activation. Furthermore, EGF-stimulated downregulation of E-cadherin and demsoglein-2 displays distinct temporal and spatial patterns and involves different mechanisms. These findings indicate that the same stimulus leads to different outcomes for classical versus desmosomal cadherins in keratinocytes.

## 2. Materials and Methods

### 2.1. Cell Line and Reagents

Squamous cell carcinoma (SCC) 12F cells were originally derived from a tumor of the facial epidermis and generously provided by Dr. William A. Toscano, Jr. (University of Minnesota, Minneapolis, MN). SCC 12F cells are nontumorigenic and express EGF receptor levels similar to those detected at the margins of healing wounds. SCC 12F cells were maintained on 10 cm^2^ plates in Dulbecco's modified Eagle's medium : Ham's F-12 nutrient mixture (DMEM : F-12) containing 5% (v/v) iron-supplemented defined calf serum (HyClone Laboratories, Inc., Logan UT), 2 mM L-glutamine, and antibiotics (penicillin, 100 U/mL, streptomycin, 50 *μ*g/mL). For all experiments involving growth factor addition, SCC 12F cells were placed into DMEM : F-12 containing 0.1% (w/v) bovine serum albumin (BSA) for 24 hours prior to growth factor addition. Murine epidermal growth factor (EGF) was obtained from Biomedical Technologies Inc. (Stoughton, MA). GM6001X was purchased from Chemicon (Temecula, CA). AG1478 was purchased from Enzo Life Sciences (Plymouth Meeting, PA). DMEM : F-12, BSA, Penicillin/Streptomycin, and L-glutamine were purchased from Sigma Chemical Co. (St. Louis, MO).

### 2.2. Immunohistochemistry

129 Young adult male mice were shaved and depilated using Nair (Church and Dwight, Princeton, NJ). Two days later, mice were anesthetized, skin of the upper dorsum was tented, and paired 3 mm-diameter excisional wounds were introduced using a sterile disposable biopsy punch. Mice were sacrificed by CO_2_ inhalation 48 hours later. Skin containing the wound sites and underlying muscle was removed, spread on thin cardboard, fixed in 10% neutral buffered formalin, embedded in paraffin, and sectioned at 4 *μ*m. For immunohistochemistry, sections were deparaffinized and rehydrated. Endogenous peroxidase was blocked by a 10-minute incubation in aqueous 3% H_2_O_2_, and microwave antigen retrieval was performed in 10 mM citrate buffer, pH 6.0. Nonspecific antibody binding was blocked using Biocare Blocking Reagent (Concord, CA). For E-cadherin immunohistochemistry, slides were incubated for 1 hour in a 1 : 50 dilution of a rabbit polyclonal antibody (sc7870, Santa Cruz, Santa Cruz, CA), then for 30 minutes with Envision plus labeled polymer-anti-rabbit-HRP (Dako, Carpinteria, CA). Desmoglein was detected using a mouse monoclonal antibody (CBL174, Millipore, Billerica, MA) diluted 1 : 100 for a 1-hour incubation, followed by a 15-minute incubation with biotinylated rabbit-anti-mouse F(ab)' (Accurate Chemical, Westbury, NY) diluted 1 : 250 and a 30-minute incubation with SA-HRP (BioGenex, San Ramon, CA). All incubations were performed at room temperature, and immunoreactivity was detected using diaminobenzidine as chromagen. Slides were counterstained with hematoxylin, dehydrated, and cover-slipped.

### 2.3. *In Vitro* Wound Healing Assay

For evaluation of *in vitro *reepithelialization, confluent cell monolayers were deprived of serum and growth factors for 24 hours, and a cell-free area was introduced by scraping the monolayer with a standard dimension blue pipette tip (USA Scientific, Ocala, FL) followed by extensive washing to remove cellular debris. *In vitro *reepithelialization was monitored by repopulation of the cleared area (wound width typically between 200–300 mm) with cells over time either in the presence or absence of the EGF receptor inhibitor AG1478.

### 2.4. Immunoblotting of Protein and Conditioned Medium

Cells were lysed either with SDS collection buffer (10 mM Tris pH 7.5, 1% SDS, 5 mM EDTA, 2 mM EGTA, 1 mM PMSF, 1 *μ*g/mL leupeptin, 1 *μ*g/mL pepstatin A) to collect total protein, or by subcellular fractionation. For subcellular fractionation, cells were lysed with 0.05% saponin collection buffer (10 mM Tris pH 7.5, 140 mM NaCl, 0.05% saponin, 5 mM EDTA, 2 mM EGTA, 1 mM PMSF, 1 *μ*g/mL leupeptin, 1 *μ*g/mL pepstatin A) and then centrifuged at 14,000 rpm; the resulting supernatant represented the saponin, or cytoplasmic, fraction. The pellet was then fully resuspended in 1% triton collection buffer (10 mM Tris pH 7.5, 140 mM NaCl, 1% Triton X-100, 5 mM EDTA, 2 mM EGTA, 1 mM PMSF, 1 *μ*g/mL leupeptin, 1 *μ*g/mL pepstatin A) and then centrifuged, and the resulting supernatant was labeled the triton soluble, or membrane-associated fraction. Finally, the remaining pellet was fully resuspended in 1% SDS collection buffer, and this was labeled the triton insoluble, or cytoskeletal-associated (junction bound) fraction. Protein concentrations were determined by bicinchoninic acid (BCA) colorimetric assay (Pierce, Rockford, IL). Equal quantities of protein were fractionated on 8% (w/v) SDS polyacrylamide gels and transferred onto polyvinylidene fluoride (PVDF) membranes. Conditioned medium was collected from 6 well plates (1 mL total volume) and then concentrated using a centrifugal filter device (Millipore, Bedford, MA) with a 30,000 molecular weight cutoff. The retentate was collected and resolved on an 8% (w/v) SDS polyacrylamide gel, then transferred onto a polyvinylidene fluoride (PVDF) membrane. Immunoblotting was performed as described below. Membranes were blocked in 5% (w/v) nonfat dry milk in Tris-buffered saline with 0.05% Tween-20 (TBST) for 1 hour at room temperature before the addition of primary antibody. Primary antibodies [E-cadherin (clone NCH-38, Dakocytomation, Carpinteria, CA), E-cadherin (Santa Cruz Biotechnology, Santa Cruz, CA), desmoglein-2 (clone 6D8, Invitrogen, Carlsbad, CA), and alpha-catenin (Chemicon, Temecula, CA)] for western blots were used at 1 : 1000 dilution and incubated at room temperature for 1 hour. The membranes were washed with TBST three times, and secondary antibody horseradish peroxidase labeled goat anti-mouse from Promega (Madison, WI), at a dilution of 1 : 10,000 in 5% (w/v) milk was added for 1 hour at room temperature. The membranes were washed with TBST three times and developed using the SuperSignal chemiluminescent detection system (Pierce, Rockford, IL). Visualization and densitometry of the blots were obtained with the Kodak Image Station 440 System (New England Nuclear, Boston, MA).

### 2.5. Immunofluorescence

SCC 12F cells were seeded in a Lab Tek II chamber slide system (Nalge Nunc International, Naperville, IL). Cells were transferred to serum-free medium and then treated with 20 nM EGF for various times, or pretreated with the broad spectrum MMP inhibitor GM6001X for 30 minutes prior to addition of EGF. To probe for junctional proteins, cells were fixed with cold dry methanol for 2 minutes, or alternatively with freshly prepared paraformaldehyde (3.7%) for 10 minutes. Paraformaldehyde slides were triton-permeabilized for an additional 5 minutes. The slides were then blocked in 3% (w/v) BSA in complete PBS (phosphate-buffered saline containing 0.8 mM magnesium chloride and 0.18 mM calcium chloride) at 37°C in a humidified chamber. For detection of lysosomal colocalization, Lysotracker (Invitrogen, Carlsbad, CA) was added at a 1 : 250 dilution to live cells 2 hours before fixation. Primary antibodies were used at a 1 : 100 dilution and included: beta-catenin (Chemicon, Temecula, CA), plakoglobin, caveolin-1, pancytokeratin (Santa Cruz, Santa Cruz, CA), E-cadherin (HECD-1 clone), desmoglein-2 (clone 6D8, Invitrogen, Carlsbad CA), EEA1 (Affinity Bioreagents, Golden, CO), epidermal growth factor receptor (Upstate, Chicago, Il.), rab11 (rabbit polyclonal Ab), and rab7 (chicken polyclonal Ab), both kindly provided by Dr. Angela Wandinger-Ness, University of New Mexico. Primary antibody was incubated for 1 hour at 37°C in a humidified chamber. Slides were washed three times in complete PBS; then a fluorophore-conjugated secondary antibody (Invitrogen, Carlsbad, CA) was added at a 1 : 300 dilution and was allowed to incubate for 1 hour at 37°C in a humidified chamber. Actin staining was obtained by incubating with TRITC-labeled Phalloidin (0.5 *μ*g/mL, Sigma, St. Louis, MO) for 30 minutes at room temperature. Slides were washed three times in complete PBS then mounted with a coverslip using Vectashield mounting medium (Vector Labs, Burlingame, CA). Images were obtained with an inverted microscope (Olympus IX70, Melville NY) and MagnaFire software 2.1 (Optronics, Goleta, CA) or with a Zeiss confocal Microscope (Zeiss, Thornwood, NY).

### 2.6. PCR

RNA was isolated using 0.5 mL Trizol (Invitrogen Life Technologies, Carlsbad, CA), following the manufacturer's instructions. cDNA was synthesized from total RNA. All PCR reagents were purchased from Promega (Madison, WI). Primers were ordered from Sigma-Genosys (The Woodlands, TX) and included the following: E-cadherin (Forward, GGGTGACTACAAAATCAATC, Reverse, GGGGGCAGTAAGGGCTCTTT), desmoglein-2 (Forward, CACTATGCCACCAACCACTG, Reverse, TTAGGCATGGCCAGAGTAGG), and 18s rRNA (Forward, AAACGGCTACCACATCCAAG, Reverse, CCTCCAATGGATCCTCGTTA). E-cadherin amplification was performed with an initial denaturation step at 94°C for 4 minutes, followed by 38 cycles with a denaturing step at 94° for 30 seconds, an annealing step of 55°C for 30 seconds and an extension step of 72°C for 30 seconds, and a final extension step of 72°C for 4 minutes. Desmoglein-2 amplification was performed as above, with the annealing step of 64°C for 30 seconds, for a total of 30 cycles. 18s rRNA amplification was performed with an initial denaturation step at 94°C for 4 minutes, followed by 20 cycles with a denaturing step at 94° for 30 seconds, an annealing step of 55°C for 30 seconds and an extension step of 72°C for 30 seconds, and a final extension step of 72°C for 4 minutes. GoTaq Flexi products (Promega, Madison, WI) were used according to the manufacturer's protocols. The resulting PCR products were loaded onto a 3% agarose gel (EMD Chemicals, San Diego, CA). SYBR Safe DNA gel stain (Invitrogen, Carlsbad, CA) was added for staining purposes, and the gel was imaged on a Kodak Image Station 440 System (New England Nuclear, Boston, MA).

### 2.7. Statistical Analysis

Results from different treatment groups in immunoblotting experiments were compared by Welsh's *t*-test, and the value for statistical significance was considered at *P* < 0.05.

## 3. Results

### 3.1. Wound Margins *In Vivo* Show a Decrease in E-Cadherin and Desmoglein at the Migrating Epithelial Tip

**Figure 1:**
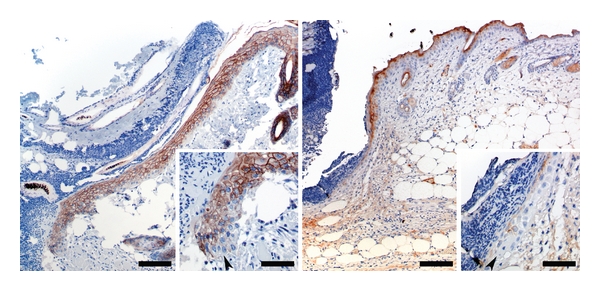
Cadherin staining 48 h postincisional wounding *in vivo*. Left Panel: E-cadherin immunoreactivity at wound margin 48 h after introduction of an incisional wound. Bar = 150 *μ*m. Inset: Higher magnification of the advancing edge of epithelium. Note that E-cadherin immunoreactivity is present in all but a few cells at the very tip of the migrating epithelium. Bar = 50 *μ*m. Right Panel: Desmoglein immunoreactivity at wound margin 48 h after introduction of an incisional wound. Bar = 150 *μ*m. Inset: Higher magnification of the advancing edge of epithelium. Note that desmoglein immunoreactivity is largely absent from the tip of the migrating epithelium. Bar = 50 *μ*m.

Both E-cadherin and desmoglein were studied in an *in vivo* incisional wound model. After 48 hours after incision, the epithelium stained for both E-cadherin (Figure 1, left panel) and desmoglein (Figure 1, right panel), throughout a majority of the intact epithelium. However, at the migrating wound edge, both E-cadherin and desmoglein displayed a marked decrease in staining (Figure 1, insets of both panels, E-cadherin was present in all but a few cells at the migrating tip, demonstrating the persistence of this cadherin throughout the epithelium even 48 hours post injury. Desmoglein, however, is largely absent from the migrating front. Clearly, a downregulation of both cadherins occurs as a normal response to wounding *in vivo*, although the degree of downregulation of each cadherin differs 48 hours post injury.

### 3.2. EGF Receptor Activation Is Required for Junctional Disruption at Wound Margins

**Figure 2:**
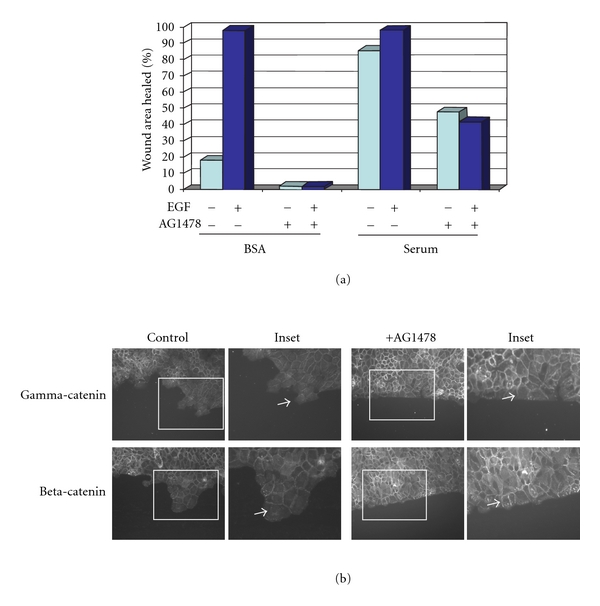
Junctions at the wound margin are retained upon EGF receptor inhibition. Cells were grown to confluence, placed in serum-free medium overnight, and then a wound was introduced with a pipette tip. Cells were washed 3X with PBS, then placed in serum-free medium +/−20 nM EGF and +/−5 *μ*M of the selective EGF receptor inhibitor AG1478 as indicated for 24 h. (a) The area of wound closure was measured using ImagePro software. (b) After wounding, cells were fixed and immunostained for either gamma-catenin (plakoglobin) or beta-catenin. Note the loss of border staining is limited to the wound edge (see arrows). Upon EGF receptor inhibition, junctional proteins are apparent at the wound edge (see arrowheads).

EGF receptor activation stimulates wound repair *in vivo* [30, 40] and disrupts junctional complexes *in vitro *[41, 42]. We conducted *in vitro* wound closure assays using SCC 12F cells, a well-differentiated squamous cell carcinoma line [43] with EGF receptor levels comparable to those at wound margins [36] to investigate the status of junctional complexes at an *in vitro* wound border as a function of EGF receptor activity (Figure 2). Modest wound closure occurs in serum-free conditions, and exogenous EGF greatly stimulates the response. Both basal and EGF-stimulated migration into the wound area was inhibited by the EGF receptor tyrosine kinase inhibitor AG1478 (Figure 2(a)). The loss of basal migration is likely due to inhibition of the autocrine EGF receptor activation in this system [44]. Junctional disruption was detected at wound margins as evidenced by the loss of catenin border staining under basal (no exogenous EGF) conditions (Figure 2(b), arrows). In contrast, EGF receptor inhibition led to retention of catenin staining at cell borders of cells at the wound edge, suggesting that junctional integrity was retained (Figure 2(b), arrowheads). Similar results were obtained for E-cadherin and desmoglein-2 (data not shown).

### 3.3. Downregulation of Junctional Proteins by EGF Receptor

**Figure 3:**
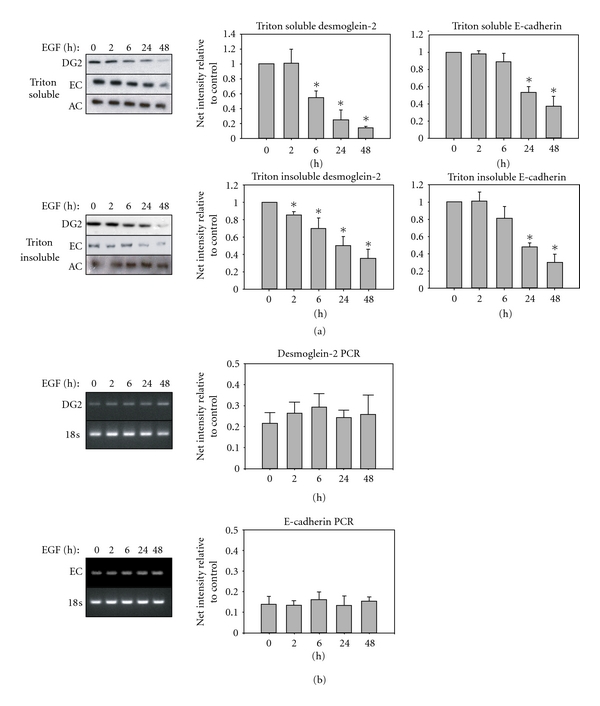
EGF regulation of cadherin proteins in membrane and cytoskeleton-associated pools. Cells were grown to subconfluence, placed in serum-free medium overnight, and then treated with EGF for the indicated times. DG2: desmoglein-2, EC: E-cadherin, AC: alpha-catenin. (a) Sequential detergent extraction separates the triton soluble (membrane-associated) fraction, from the triton insoluble, (cytoskeletal, or intact junctional) fraction. Blots are representative of a minimum of three separate experiments. Bar graphs represent the densitometric quantification of each lane normalized to no treatment control, with asterisks indicating statistical significance. (*P* < 0.05) (b) Cells were treated with or without EGF for the indicated times, and mRNA level was measured by PCR. Bar graphs represent the densitometric quantification of bands normalized to no treatment control.

EGF receptor activity has been reported to modulate certain junctional proteins, leading to downregulation and protein degradation [9, 45–47]. The catenins are well studied, but less is known regarding the fate of cadherins in response to EGF receptor activation. Using sequential detergent extraction, we compared protein levels of membrane-associated (triton soluble) versus junction bound (triton insoluble) cadherins. Extended exposure to EGF led to loss of both the desmosomal cadherin desmoglein-2 and adherens junction protein E-cadherin from the cell membrane and junction-associated protein pools (Figure 3). Since transcriptional repression of E-cadherin is one reported mechanism of down-regulation [48, 49], we examined mRNA levels for E-cadherin and desmoglein-2 in response to EGF (Figure 3(b)). In a time course that spanned 48 hours, no change in either E-cadherin or desmoglein-2 transcripts occurred. In agreement with the *in vitro* wound model, a decrease in the protein levels of both beta-catenin and plakoglobin was detected in the membrane- and junction-associated protein fractions (data not shown). Interestingly, not all junctional components were affected, as the levels of the adherens junctional linker protein alpha-catenin were retained following EGF treatment (Figure 3(a)).

There was a notable difference in the time dependence for loss of cadherin protein in the membrane-associated versus junction-associated protein pools in response to EGF. The triton soluble, membrane-associated fraction, showed a decrease in desmoglein-2 protein as early as 6 hours, whereas E-cadherin was not significantly decreased until 24 hours after EGF exposure (Figure 3(a), asterisks *P* < 0.05). Similarly, in the triton insoluble fraction, a significant decrease in desmoglein-2 protein was evident at 2 hours post-EGF treatment whereas little change in E-cadherin was evident until later time points (Figure 3(b), asterisks *P* < 0.05). These findings indicate that E-cadherin and desmoglein-2 are not coordinately regulated during reepithelialization.

### 3.4. EGF-Dependent Downregulation of Desmoglein-2 Precedes E-Cadherin

**Figure 4:**
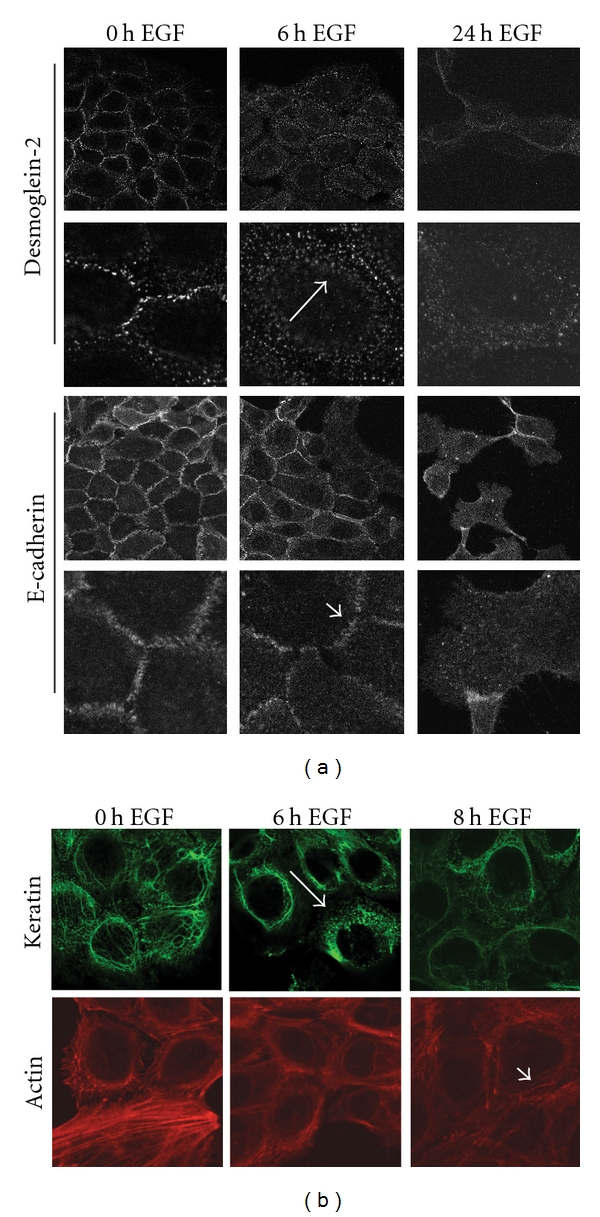
Different kinetics for disruption of desmosomes and adherens junctions by EGF. (a) SCC 12F cells were treated with EGF for the indicated times, fixed, and then probed with E-cadherin or desmoglein-2 antibodies. Note the relocalization of the desmosomal cadherin desmoglein-2 from the cell borders following treatment with EGF for 4–6 h (white arrows). This pattern differs from that observed for the adherens junctional cadherin, E-cadherin, where strong border staining is evident at 6 hours post EGF treatment (white arrowheads). (b) Cells were treated with EGF, fixed, and then stained with phalloidin to stain the actin cytoskeleton, or with a pan-cytokeratin antibody, to label keratin filaments. Disorganization of the keratin network is seen at 6 hours (white arrow), while the actin cytoskeleton remains intact at 8 hours posttreatment (white arrowheads).

The cellular localization of E-cadherin and desmoglein-2 following EGF receptor activation confirmed the differences observed in the cell fractionation studies. Internalization of desmoglein-2 was evident within 6 hours of EGF treatment as detected by punctate intracellular staining (Figure 4(a), white arrows). In contrast, E-cadherin staining was retained at the cell borders after EGF treatment (Figure 4(a), arrowheads). Loss of both cadherins from cell borders was evident after 24 hours of EGF treatment. Similar internalization kinetics were observed for the desmosomal catenin plakoglobin and the adherens junctional component, beta-catenin (data not shown).

Immunostaining of the corresponding cytoskeleton partners shows disruption of the desmosomal-associated cytokeratin network at 6 hours, consistent with the time frame of desmosomal cadherin internalization (Figure 4(b)). In contrast, the adherens junction-associated actin cytoskeleton remains intact at this timepoint and is maintained at 8 hours following EGF treatment, consistent with E-cadherin localization. Disruption of the actin skeleton was evident 24 hours posttreatment (data not shown). These findings indicate that functional disruption of desmosomes precedes that of adherens junctions.

### 3.5. Internalization Fate of Desmoglein-2

**Figure 5:**
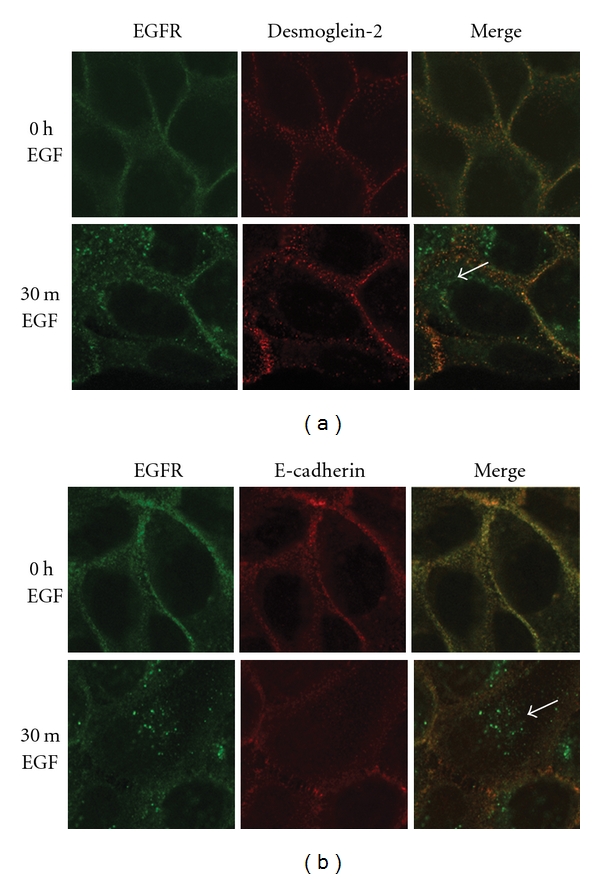
Cadherins do not cointernalize with the EGF receptor. SCC 12F cells were treated with 20 nM EGF for 30 minutes, fixed, and probed for EGF receptor (green) or junctional cadherin (red). White arrows indicate the internalized EGF receptor in the cytoplasm while both junctional cadherins, desmoglein-2 (upper panel), and E-cadherin (lower panel) remain at the cell surface 30 minutes post EGF treatment.

**Figure 6:**
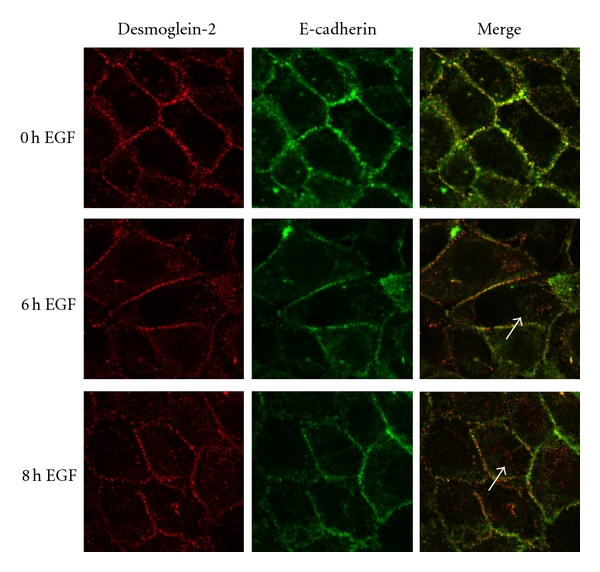
Desmoglein-2 does not internalize with E-cadherin. SCC 12F cells were treated with EGF for the indicated times, fixed, then probed with E-cadherin or desmoglein-2 antibodies. The desmosomal cadherin desmoglein-2 is relocalized from the cell borders at 6–8 hrs after EGF treatment. Note punctate cytoplasmic staining (white arrows). This pattern differs from that observed for the adherens junctional cadherin, E-cadherin, where strong border staining is evident at 6 hours post EGF treatment.

Several studies document E-cadherin internalization in response to growth factors [19, 50–52]; however, EGF-stimulated trafficking of desmosomal cadherins has not been well described. EGF receptor activation stimulates multiple trafficking pathways including clathrin-dependent and –independent trafficking itineraries [19, 53, 54]. EGF stimulates clathrin-dependent EGF receptor internalization [55, 56], although the receptor can undergo clathrin-independent internalization under certain situations such as oxidative stress [57]. Neither E-cadherin nor desmoglein-2 colocalized with the EGF receptor following EGF receptor activation, and EGF receptor internalization preceded that of desmoglein-2 by several hours (Figure 5, white arrows). These findings indicate that although desmoglein-2 internalizes in response to EGF receptor activation, the two proteins do not follow the same itinerary. Dual immunofluorescence staining for desmoglein-2 and E-cadherin reveals that desmoglein-2 colocalizes with E-cadherin at the cell surface, but not in the cytosol post EGF treatment (Figure 6) further indicating distinct fates for the two cadherins.

**Figure 7:**
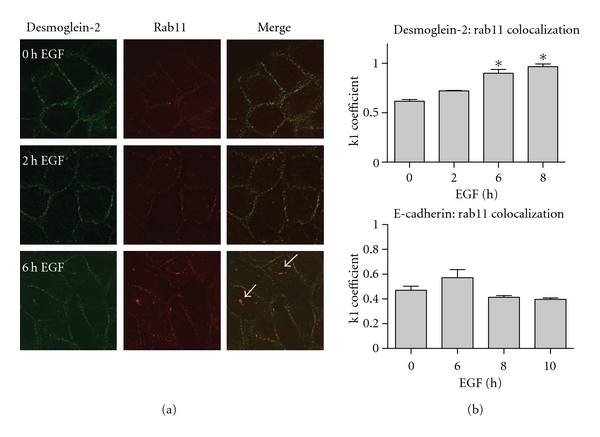
Desmoglein-2 colocalizes with Rab11, a recycling marker. (a) SCC 12F cells were treated for the indicated times with 20 nM EGF, then fixed with 3.7% formaldehyde and permeabilized with 0.1% Triton X-100. Cells were stained with antibodies against both desmoglein-2 and rab11. Secondary antibodies tagged with either FITC or Rhodamine were used. Colocalization is detected by the appearance of yellow staining where the red and green overlap, particularly in EGF-treated cells (white arrows). (b) Mandler's overlap coefficient, k1, measures the ratio of cadherin colocalized with Rab11 to total cadherin present.

To establish whether desmoglein-2 internalization occurs through the classical endosomal pathway or through alternate internalization pathways, we used markers of the classical endosomal pathway (EEA-1, Rabs) as well as markers for caveolae-dependent internalization (caveolin-1). A small fraction of desmoglein-2 was present in EEA-1-positive early endosomes. However, there was little evidence of desmoglein-2 colocalizing with rab7, a late endocytic vesicle marker, nor with lysotracker, a marker of lysosomes, for up to 12 hours post EGF treatment (summarized in Table 1). Similarly, desmoglein-2 did not colocalize with caveolin-1, a marker for the caveosomal-dependent trafficking. The majority of desmoglein-2 colocalized with the recycling marker Rab11 (Figure 7(a)). Colocalization was analyzed using Mander's overlap coefficients k1 and k2 [58], where the k1 coefficient described the amount of cadherin colocalized with Rab11 as compared to total cadherin levels. Over an 8-hour time course, desmoglein-2 colocalization with Rab11 increased (Figure 7(b)) and at 6- and 8-hour time points, both proteins were present in small punctate vesicles on the plasma membrane and in the cytosol. This colocalization was statistically significant at the 6- and 8-hour time points (Figure 6(b), *P* < 0.05) whereas E-cadherin showed no increase in k1 over the same timepoints. Together, these findings indicate that EGF promotes desmoglein-2 internalization through a predominantly recycling rather than a lysosomal degradation pathway and represents a novel internalization fate for desmoglein-2.

### 3.6. EGF Receptor Activation Stimulates E-Cadherin Cleavage

**Figure 8:**
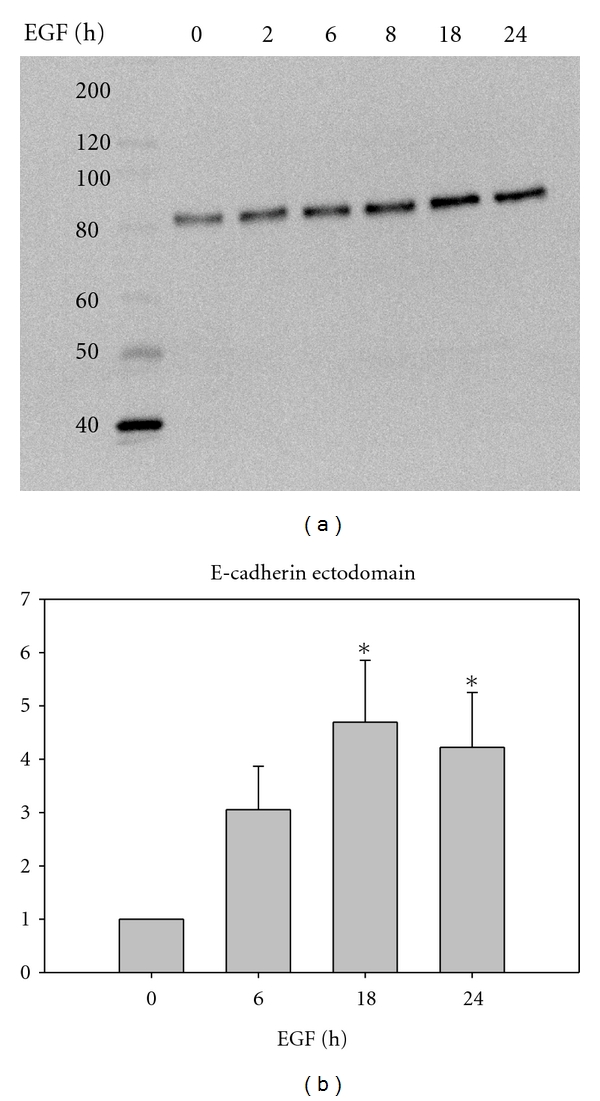
EGF receptor activation leads to accumulation of an E-cadherin extracellular fragment in conditioned medium. Subconfluent cells were serum-starved (without BSA in the medium) for 24 hours. Cells were treated with 20 nM EGF for the indicated times, then the conditioned medium was collected, concentrated, and resolved on an 8% SDS gel. Samples of conditioned media were normalized to the corresponding protein lysate, transferred onto PVDF, and probed with an antibody against the extracellular epitope of E-cadherin, which recognizes both full length (120 kD) and cleaved E-cadherin (80 kD).

**Figure 9:**
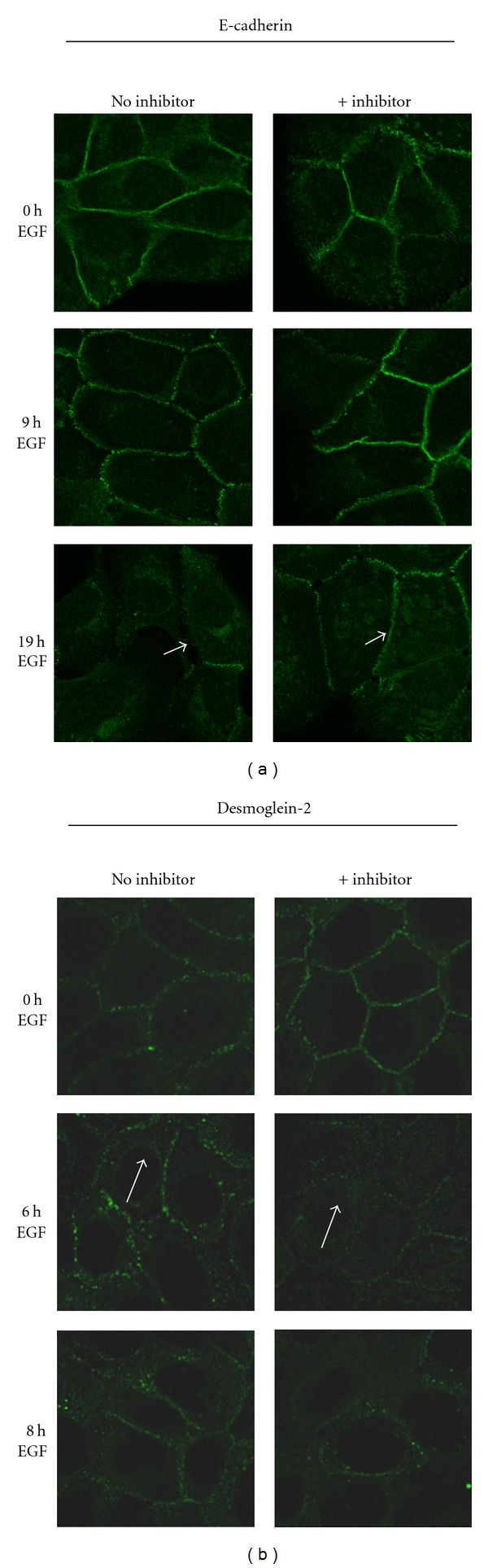
Effect of MMP inhibition on membrane localization of E-cadherin or desmoglein-2 after EGF treatment. Cells were serum-starved for 24 hours then treated with 50 *μ*M of the broad spectrum MMP inhibitor GM6001X for 30 minutes before addition of 20 nM EGF for the indicated times. Cells were then fixed and probed with either an E-cadherin antibody that recognizes the intracellular epitope or desmoglein-2 antibody. White arrows indicate the internalized desmoglein-2 even in the presence of inhibitor, while the white arrowheads indicate the retention of E-cadherin at the cell-cell borders.

In contrast to desmoglein-2, EGF treatment led to a gradual decrease in the intensity of E-cadherin staining at cell-cell borders without an emergence of punctate intracellular staining indicative of internalization after 8-hour treatment (see Figure 6). However, at these same timepoints, E-cadherin cleavage as a consequence of EGF receptor activation was detected by immunoblotting for the 80 kD E-cadherin ectodomain. A significant increase in an 80 kD E-cadherin fragment in the conditioned medium was evident 18 hours and 24 hours after EGF treatment (Figure 8). There was no evidence for desmoglein-2 cleavage products under the same conditions (data not shown). Accumulation of the EGF-dependent 80 kD E-cadherin fragment was prevented by pretreatment with a broad-spectrum matrix metalloproteinase (MMP) inhibitor GM6001X. Although there is some internalized E-cadherin after 18 hours of EGF treatment, cells pretreated with the MMP inhibitor retain significant junctional E-cadherin staining at the plasma membrane (Figure 9). Conversely, this protection was not extended to the desmosomal cadherin desmoglein-2, as internalization was independent of the MMP inhibitor. Taken together, these studies indicate distinct fates for the desmosomal cadherin desmoglein-2 as compared to the classical cadherin E-cadherin upon EGF stimulation.

## 4. Discussion

The EGF receptor is a significant regulator of cutaneous wound repair based on experimental studies, genetic models, and evidence for accelerated healing in patients [28–30]. In this study we demonstrate temporal differences in EGF-dependent down-regulation of E-cadherin and desmoglein-2. Our studies demonstrate a decrease of desmosomal function precedes that of adherens junctions *in vivo *and for the EGF-stimulated responses *in vitro*. This finding is consistent with previous reports that assembly of adherens junctions is necessary for the formation of desmosomes [59]. The potential significance of desmosomal disruption preceding that of adherens junctions may be related to the requirement of keratinocytes to form a migratory epithelial sheet from the stratified epidermis [1]. It has been noted that conversion to a single cell layer requires down-regulation of desmosomal adhesion [60]. Importantly, we provide evidence that the EGF-stimulated disruption of the two types of junctions is related to different underlying mechanisms for the down-regulation of cadherin function.

EGF receptor activation led to accumulation of an 80 kD E-cadherin fragment in conditioned medium, and a broad-spectrum MMP inhibitor protected adherens junctions from EGF-dependent disruption. Extracellular cleavage of E-cadherin is a well-studied mechanism, and several proteases, including MMP-3, -7 [18], -9 [16], MT1-MMP, ADAM10 [61] and ADAM15 [62], as well as plasmin [17], kallikrein 7 [63], and *γ*-secretase [64, 65] cleave E-cadherin. EGF is a known inducer of MMPs and other proteases, and there is evidence for EGF-stimulating MMP-dependent E-cadherin cleavage [16, 35, 66–71]. Although the internalization of E-cadherin has been reported in several models and occurs through clathrin-dependent [24, 25, 50, 72–74], clathrin-independent [75, 76], and caveolae-dependent [54] pathways, the endocytic fate of E-cadherin appears to vary according to the stimulus presented and cell context [54, 77, 78]. Src activation promotes clathrin-dependent endocytosis of E-cadherin [25], yet clathrin-independent mechanisms were reported following EGF stimulation of the MCF-7 and A431 cell lines by micropinocytosis [19] and caveolar-dependent internalization [54], respectively. In contrast to studies demonstrating cointernalization of E-cadherin with the tyrosine kinase receptors c-Met and FGFR1 [50, 51], we did not find evidence for concurrent trafficking of EGF receptor with either E-cadherin or desmoglein-2.

Similar to E-cadherin, extracellular cleavage of desmosomal cadherins has been reported. The appearance of a 60 kD fragment of desmoglein-3 occurs *in vitro* in keratinocytes treated with patient sera with pemphigus vulgaris [15]. In a highly invasive squamous cell line that forms sparse cell : cell junctions, a 100 kD desmoglein-2 fragment was detected in low-calcium (.09 mM) conditions, and production of this fragment was reversed by inhibition of the EGF receptor and the inhibition of several members of the sheddase family of ADAMS (a disintegrin and metalloprotease), including ADAM17 [79]. ADAM 17 has been shown by others to be increased in response to EGF [80] and to cleave desmoglein-2 [81]. Our studies suggest that EGF receptor-stimulated desmoglein-2 cleavage is not the predominant mechanism in this experimental system.

There is additional evidence that desmosomal cadherins can be internalized in response to various stimuli. In HaCat cells, desmoglein-1 underwent internalization in response to sera collected from pemphigus vulgaris and pemphigus foliaceus patients as early as 6 hours with a concurrent decrease in the amount of desmoglein-1 found in the membrane-associated fraction postexposure [21]. Internalization of desmoglein-3 in response to pemphigus autoantibody was found to undergo clathrin-independent internalization [22], early endosomal localization [23], and subsequent lysosomal degradation [20]. Both EGF receptor-dependent and -independent mechanisms have been proposed [82–84].

The potential significance of reversible mechanisms for downregulation of cadherins may be related to characteristics of the partial EMT present at wound margins [1]. There has been increased appreciation that partial EMT includes processes of dynamic junctional modulation and retention of cell cohesion rather than complete cell dissociation and migration of individual cells. As examples, both in culture and *in vivo*, maintenance of cell : cell contact of migrating neural crest cells is necessary for movement persistence and oriented migration [85]. In the Drosophila tracheal system, dynamic modulation of junctions through endocytosis and recycling maintains tissue stability during migratory processes [86], and during gastrulation, posttranslational regulatory mechanisms such as E-cadherin protein degradation and turnover appear to be important [85]. One characteristic of cohesive migrating epithelial is that cells at the edge of these tissues appear more mesenchymal [87] as we observe at the edge of wound margins *in vitro* and *in vivo* (Figures 1 and 2).

Collectively, the data indicates that there are numerous trafficking itineraries and mechanisms of cadherin down-regulation that may be dependent upon the initial signaling event. Our studies demonstrate EGF-stimulated down-regulation of E-cadherin and desmoglein-2 through distinct mechanisms; EGF-dependent cleavage of E-cadherin and entry of desmoglein-2 into a recycling trafficking itinerary. These distinct mechanisms may help account for the observed differences in the modulation of adherens junctions and desmosomes during wound repair *in vivo.*

## 5. Conclusions

Modulation of adherens junctions and desmosomes is an essential component of reepithelialization, yet we do not have a full understanding of the mechanisms that govern assembly and disassembly of these structures and, in particular, the fate of the respective cadherins. In this study we demonstrate that EGF downregulates E-cadherin and desmoglein-2, but with different kinetics and through distinct mechanisms. Activation of the EGF receptor leads to E-cadherin cleavage through an MMP-dependent mechanism. In contrast, a desmosomal cadherin, desmoglein-2, is internalized and localizes predominantly within a recycling compartment rather than trafficking to lysosomes, thereby representing a novel fate in response to growth factor activation. This study illustrates that the same stimulus can lead to divergent outcomes for disruption of adherens junctions and desmosomal complexes, and different fates for the corresponding cadherins. Understanding how various stimuli direct cadherin trafficking and downregulation will likely prove important to understanding junctional modulation in physiologic and pathophysiologic conditions.

## Figures and Tables

**Table 1:** Localization of cadherins with endocytic trafficking markers. Colocalization experiments were conducted using immunofluorescence techniques and confocal microscopy as described in “Methods”. EGF treatment ranged from 2–12 hours, and experiments were repeated a minimum of 3 times. (−) indicates no colocalization at any timepoint; (±) indicates colocalization at the plasma membrane but not in cytoplasm; (+) indicates vesicular colocalization in at least one timepoint; (+++) indicates colocalization in vesicles at several timepoints.

Endosomal compartment	Desmoglein-2 colocalization	E-cadherin colocalization
EEA1 (Early endosome)	+	N. T.
Rab11 (Recycling endosome)	+++	−
Rab7 (Late endosome)	−	−
Lysotracker (Lysosome)	+	−
Caveolin-1 (Caveosome)	+	(±)

## Abbreviations

EGF: epidermal growth factor,
MMP: matrix metalloproteinase,
EMT: epithelial-mesenchymal transition,
PCR: polymerase chain reaction,
SCC: squamous cell carcinoma,
BSA: bovine serum albumin,
PBS: phosphate buffered saline,
SDS: sodium dodecyl sulfate,
TBST: Tris buffered saline with Tween,
EDTA: Ethylenediaminetetraacetic acid,
EGTA: Ethyleneglycoltetraacetic acid,
PMSF: phenylmethanesulfonyl fluoride.
